# Knowledge and attitudes of rural healthcare providers regarding domestic violence against women: a systematic review

**DOI:** 10.1590/1516-3180.2022.0682.R1.180723

**Published:** 2023-12-04

**Authors:** Cláudio Tarso de Jesus Santos Nascimento, Maria Tereza Campos Vidigal, Vinícius Henrique Ferreira Pereira de Oliveira, Raquel Porto Alegre Valente Franco, Walbert Andrade Vieira, Adriana de-Jesus-Soares, Rafael Rodrigues Lima, Ademir Franco, Luiz Renato Paranhos

**Affiliations:** I MSc. Physical Educator, Doctoral student, Postgraduate Program in Dentistry, Universidade Ceuma, São Luís (MA), Brazil.; II MSc. Dentist, Master’s student, Postgraduate Program in Dentistry, School of Dentistry, Universidade Federal de Uberlândia, Uberlândia (MG), Brazil.; III MSc. Dentist, Master’s student, Postgraduate Program in Dentistry, School of Dentistry of Araraquara, Universidade Estadual Paulista, Araraquara (SP), Brazil.; IV MSc. Dentist, Doctoral student, Postgraduate Program in Dentistry, School of Dentistry, Universidade Federal de Uberlândia, Uberlândia (MG), Brazil.; V MSc. Dentist, Doctoral student, Department of Restorative Dentistry, Endodontics Division, School of Dentistry of Piracicaba, Universidade Estadual de Campinas, Piracicaba (SP), Brazil.; VI PhD. Dentist, Professor, Department of Restorative Dentistry, Endodontics Division, School of Dentistry of Piracicaba, Universidade Estadual de Campinas, Piracicaba (SP), Brazil.; VII PhD. Dentist, Professor, Laboratory of Functional and Structural Biology, Institute of Biological Sciences, Universidade Federal do Pará, Belém (PA), Brazil.; VIII PhD. Dentist, Professor, Division of Forensic Dentistry, Faculdade São Leopoldo Mandic, Campinas (SP), Brazil.; IX PhD. Dentist, Professor, Division of Preventive and Community Dentistry, School of Dentistry, Universidade Federal de Uberlândia, Uberlândia (MG), Brazil.

**Keywords:** Domestic violence, Intimate partner violence, Rural health services, Spouse abuse, Women, Educational background, Rural environment, Females victims

## Abstract

**BACKGROUND:**

Specific types of violence such as intimate partner sexual violence and intimate partner homicide occur more frequently in rural areas.

**OBJECTIVE:**

This study aimed to systematically review the literature on the knowledge and attitudes of rural healthcare providers regarding cases of domestic violence against women.

**DESIGN AND SETTING:**

Systematic review developed at Universidade Federal de Uberlândia.

**METHODS:**

We conducted an electronic search of six databases, which only included observational studies, regardless of the year, language, or country of publication, except for studies that used secondary data and were exclusively qualitative. Two reviewers performed the selection, data extraction, and risk of bias assessment using a specific Joanna Briggs Institute tool.

**RESULTS:**

Six studies met the inclusion criteria. All the studies had a low risk of bias. Approximately 38% of these professionals identified injuries caused by violence in patients. When asked about knowing the correct attitude to take in cases of confirmed violence, between 12% and 64% of rural healthcare providers answered positively; most of them would refer to specialized institutions and promote victim empowerment and counseling. The number of professionals with an educational background in the field ranged from 16% to 98%.

**CONCLUSIONS:**

The evident disparity across studies shows that some professionals have suboptimal knowledge and require training to adopt the correct attitude when identifying female victims of domestic violence in clinical practice.

**SYSTEMATIC REVIEW REGISTRATION:**

This systematic review was registered in the Open Science Framework Database under the registration http://doi.org/10.17605/OSF.IO/B7Q6S.

## INTRODUCTION

According to the Rural Health Information Hub,^1^ violence is exacerbated in rural areas, and social support for victims is not always available. The reasons behind this phenomenon include country-specific cultural differences, the education level of victims and perpetrators, and their socioeconomic status.^2^ Over the last decade, scientific literature on the topic has been scarce,^3,4^ especially if compared to studies in urban areas. Violence persists as official institutions and the scientific community overlook this scenario. The more vulnerable individuals are the predominant victims, such as children and women. All types of violence can grow exponentially if they occur in silence, such as in a domestic environment among intimate partners. The authors have highlighted that violence caused by an intimate partner might be the leading global cause of homicide of women.^5^ In this scenario, violence rates increase primarily because this is an underreported condition susceptible to the fear of retaliation.^6^

Specific types of violence are more frequent in rural areas, such as intimate partner sexual violence and intimate partner homicide.^7^ The different types of violence may lead to profound physical and psychological adverse effects on women, namely depression, anxiety, sleeping and eating disorders, panic attacks, and reduction of the quality of life as a consequence of sexually transmitted diseases, injuries, and trauma.^8^ For at least 25 years, healthcare providers have been promoted as vital components in the process of detecting, registering, and reporting cases of violence against women.^9^ Recent studies, however, have demonstrated that these professionals need more knowledge and training to identify and manage cases of violence against women.^10,11^ A systematic literature review among oral healthcare providers, for instance, revealed that less than 24% knew how to identify signs of domestic violence against women^11^. Nurses and midwives, however, seem to have a better understanding of the signs of domestic violence.^12^ The justification of subsequent research on the topic relies on the gap of scientific evidence among healthcare providers in rural areas.

By understanding the reality of rural healthcare providers and their knowledge and attitudes toward domestic violence against women, protective strategies for patients could be designed and incorporated into the routine of health services.

## OBJECTIVE

This systematic literature review compiled and analyzed evidence to understand the level of knowledge and attitudes of rural healthcare providers related to cases of domestic violence against women. To this end, the following question will be answered: “What are the knowledge and attitudes of rural healthcare providers regarding domestic violence against women?”.

## METHODS

### Protocol and registration

The protocol was reported in accordance with the Preferred Reporting Items for Systematic Review and Meta-Analysis Protocols (PRISMA-P)^13^ and registered in the Open Science Framework database (https://doi.org/10.17605/OSF.IO/B7Q6S). This systematic review was conducted according to the PRISMA^14^ and was conducted according to the Joanna Briggs Institute (JBI) Manual.^15^

### Research Question and Eligibility Criteria

The research question “What are the knowledge and attitudes of rural healthcare providers regarding domestic violence against women?” was structured with the following PICo^14^ framework: Population (P)—rural healthcare providers (doctors and nurses), Interest (I)—educational background, management, perception, knowledge level and attitude regarding cases of domestic violence against women, and Context (Co)—domestic violence against women in the rural area. The systematic review included only observational cross-sectional, cohorts, and case-control studies. No restriction of language and year of publication was applied. The exclusion criteria consisted of studies that used secondary data, such as epidemiological investigations from existing databases, surveys with questionnaires that did not include specific questions regarding violence against women in rural areas, and exclusively qualitative studies.

### Sources of information, search, and selection of studies

An electronic search was performed using MedLine/PubMed, Scopus, LILACS, SciELO, Embase, and Web of Science databases. Google Scholar, OpenGrey, and OATD were used to retrieve grey literature. Medical Subject Headings (MeSH), Health Sciences Descriptors, and Embase Subject Headings were used in their inherent databases. Synonyms and alternative terms were added to enhance the search strategy. The combination of terms was accomplished with the Boolean operators AND and OR (**Table 1**). The search was conducted in December 2021. The detected files were imported into EndNote Web (Thomson Reuters, Toronto, Canada) to remove automated duplicates. Grey literature was listed in Microsoft Word (Microsoft™ Ltd., Washington, USA) to manually remove duplicates. Prior to selecting the studies, training sessions were conducted between the two reviewers. In this phase, eligibility criteria were discussed and applied to 20% of the sample. The reviewers were considered able to proceed to the analysis of the total sample only when their agreement was ≥ 0.81 (Kappa).

**Table 1.** Strategies for database search

Database	Search Strategy (December, 2021)
**Main Databases**	
**Embase** http://www.embase.com	#**1** ‘perception’/exp OR ‘perception’ OR ‘management’/exp OR ‘management’ OR ‘sensation’/exp OR ‘sensation’ OR ‘diagnosis’/exp OR ‘diagnosis’ OR ‘knowledge’/exp OR ‘knowledge’ OR ‘attitude’/exp OR ‘attitude’ OR ‘attention’/exp OR ‘attention’
	#**2** ‘domestic violence’/exp OR ‘domestic violence’ OR ‘partner violence’/exp OR ‘partner violence’
	#**3** ‘women health’ OR ‘female’/exp OR ‘female’
	#**4** ‘health service’/exp OR ‘health service’ OR ‘medical profession’/exp OR ‘medical profession’
	**#1 AND #2 AND #3 AND #4**
**LILACS** https://lilacs.bvsalud.org/	#**1** (MH: perception OR MH: attitude OR MH: management OR MH: sensation OR MH: diagnosis OR MH: knowledge OR MH: attention)
	#**2** (MH: “domestic violence” OR “intrafamily violence” OR MH: “Intimate Partner Violence” OR MH: “Spouse Abuse”) #**3** (MH: “Women” OR MH: “Women’s Health Services” OR MH: female) #**4** (MH: “health personnel” OR “healthcare” OR MH: “Health Occupations” OR “healthcare provider”)
	**#1 AND #2 AND #3 AND #4**
**PubMed** http://www.ncbi.nlm.nih.gov/pubmed	#**1** (perception [MeSH Terms] OR attitude [MeSH Terms] OR management [MeSH Terms] OR sensation [MeSH Terms] OR diagnosis [MeSH Terms] OR knowledge [MeSH Terms] OR attention [MeSH Terms])
	#**2** (“domestic violence” [MeSH Terms] OR “intrafamily violence” [tw] OR “Intimate Partner Violence” [MeSH Terms] OR “Spouse Abuse” [MeSH Terms])
	#**3** (“Women” [MeSH Terms] OR “Women’s Health Services” [MeSH Terms] OR female [MeSH Terms])
	#**4** (“health personnel” [MeSH Terms] OR “healthcare” [tw] OR “Health Occupations” [MeSH Terms] OR “healthcare provider” [tw])
	**#1 AND #2 AND #3 AND #4**
**SciELO** https://scielo.org/	#**1** (“domestic violence” OR “intrafamily violence” OR “Intimate Partner Violence” OR “Spouse Abuse”)
	#**2** (“health personnel” OR “healthcare” OR “Health Occupations” OR “healthcare provider”)
	**#1 AND #2**
**Scopus** http://www.scopus.com/	#**1** TITLE-ABS-KEY (perception OR attitude OR management OR sensation OR diagnosis OR knowledge OR attention) #**2** TITLE-ABS-KEY “domestic violence” OR “intrafamily violence” OR “Intimate Partner Violence” OR “Spouse Abuse” #**3** TITLE-ABS-KEY “Women” OR “Women’s Health Services” OR female #**4** TITLE-ABS-KEY “health personnel” OR “healthcare” OR “Health Occupations” OR “healthcare provider”
	**#1 AND #2 AND #3 AND #4**
**Web of Science** http://apps.webofknowledge.com/	#**1** TS=(perception OR attitude OR management OR sensation OR diagnosis OR knowledge OR attention)
	#**2** TS=(“domestic violence” OR “intrafamily violence” OR “Intimate Partner Violence” OR “Spouse Abuse”) #**3** TS=(Women OR “Women’s Health Services” OR female)
	#**4** TS=(“health personnel” OR healthcare OR “Health Occupations” OR “healthcare provider”)
	**#1 AND #2 AND #3 AND #4**
**Grey Literature**	
**OpenGrey** http://www.opengrey.eu/	((violence OR “domestic violence” OR “intrafamily violence” OR “intimate partner violence” OR “spouse abuse”) AND (female OR “women’s health services” OR women)
**Open Access Theses and Dissertations (OATD)** https://oatd.org/	(perception OR attitude OR management OR sensation OR diagnosis OR knowledge OR attention) AND (violence OR “domestic violence” OR “intrafamily violence” OR “intimate partner violence” OR “spouse abuse”) AND (female OR “women\’s health services” OR women) AND (“Health personnel” OR “health care providers” OR “health care occupations” OR “health care”)
**Google Scholar** https://scholar.google.com.br/	allintitle: (perception OR attitude OR management OR sensation OR diagnosis OR knowledge OR attention) AND (violence OR “domestic violence” OR “intrafamily violence” OR “intimate partner violence” OR “spouse abuse”) AND (female OR “women’s health services”

The Rayyan Qatar Computing Research Institute (Doha, Qatar) was used for the study selection. Initially, selection was performed based only on the titles. Next, abstracts were read and selected based on eligibility criteria. Studies that did not have abstracts were kept for the subsequent phase. In this phase, full texts were read and selected and those that were excluded were registered separately with their respective reasons. If the full texts were not available via institutional access, an international bibliographic network was activated (COMUT/IBICT). Corresponding authors were contacted via e-mail as a last resort to collect full texts. All search and selection steps were performed in pairs by independent reviewers and supervised by a third researcher.

### Data collection

Prior to data extraction, a training session was conducted following the same strategy that was applied to study selection. The reviewers extracted the following data: study identifying information (authors, year of publication, and country of the study), sample characteristics (number of participants, their sex, and time of experience), characteristics of data collection (e.g. questionnaire or interviews), and the main outcomes of the study (number of rural healthcare providers with educational background on the topic, number of professionals that screen patients for signs of violence, number of professionals that state to have knowledge to identify signs and manage situations of violence against women, and the attitude of these professionals when violence is detected), which constitute the most relevant information to interpret the conclusions of the systematic review. In the case of doubt during the data extraction process, the corresponding authors were contacted up to three times via e-mail.

### Assessment of the risk of bias

The risk of bias was assessed using the JBI Critical Appraisal Checklist for Analytical Cross-Sectional Studies.^15^ As recommended by PRISMA,^14^ two reviewers independently analyzed each eligible study to assess the risk of bias. The studies were categorized based on their percentage of positive answers for the JBI questions regarding the risk of bias.^11^ High risk of bias is when the positive answers are 49% or less. Moderate risk of bias is between 50–69% of positive answers, whereas low risk of bias is when the positive answers represent 70% or more.

### Synthesis of results

Data collection was performed in the eligible studies, and the results were presented as a narrative/descriptive synthesis. The absolute (n) and relative (%) values of the participants’ answers in each study were collected. The data quantified rural healthcare providers’ educational background, management, perception, knowledge level (e.g. participation in lectures, guided orientations and discussion meetings about the theme) and attitude (e.g. any mention of professional action due to verification of signs of violence against women, regarding cases domestic violence against women).

## RESULTS

### Study selection

**Figure 1.**
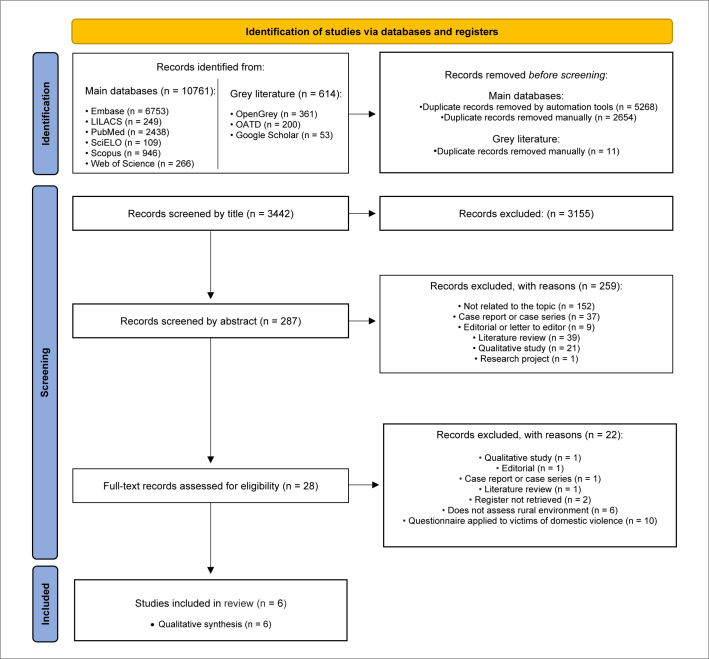
Flowchart depicting the study selection process (Preferred Reporting Items for Systematic Reviews and Meta-Analyses flow diagram).

During the first phase of study selection, 11,375 entries were identified. After removing duplicates, 3,442 entries were retained to assess titles and abstracts. After reading the titles, 3,155 entries were excluded because they did not relate to the topic. Of the 287 entries remaining for abstract reading, 259 were excluded. The remaining 28 articles were selected for full-text analysis, and 22 articles were excluded. Finally, six studies^18–23^ were included in the qualitative analysis (**Figure 1**).

### Study characteristics

The studies were published between 1998 and 2018 and performed in two different countries: four in the United States^20–23^ and two in Australia.^18,19^ All studies consisted of surveys with self-applicable questionnaires. The answers were quantified using Likert^18,20,22,23^ and adapted scales.^19,21^ All studies investigated domestic violence against intimate female partners.

Among the studies that reported the number of rural healthcare providers, 893 participants were included (705 were female). Two studies investigated the specificity of the participants^21,22^ and included family health, primary care, medical emergencies, obstetrics, and pediatrics (**Table 2**).

**Table 2.** Summary of the main characteristics of the eligible studies

Author, year and country	Sample (♂ / ♀)	Health professionals	Experience of professionals (mean in years)	Place of service	Assessment tool
Bates and Brown, 1998^18^ Australia	16 / 95	Doctors and nurses	nr	Community hospitals	Likert questionnaire
McCosker et al., 1999^19^ Australia	1 / 46	Nurses	nr	Clinics	Adapted questionnaire
Gadomski et al., 2001^20^ United States	84 / 296	nr	16	Community hospitals and clinics	Likert questionnaire
Bender, 2016^21^ United States	63 / 71	Doctors and nurses	12.2	Clinics	Adapted questionnaire
Rous and Kurth, 2016^22^ United States	13 / 75	Doctors and nurses	nr	Primary care centers	Likert questionnaire
Durham-Pressley et al., 2018^23^ United States	4 / 122 2 preferred not to inform	Nurses	18.5	Health systems hospitals	Likert questionnaire

nr = not reported in the study.

### Assessment of the risk of bias of studies

All six studies were classified as having a low risk of bias. Question 1, referring to the eligibility criteria used for sampling, was not answered in five studies.^18–21,23^ This question is relevant because it enables sample standardization and reduces the risk of bias. Questions 5 and 6 were not applicable because they referred to experimental studies on exposure or interventions. All remaining questions had positive answers in all studies (**Table 3**).

**Table 3.** Risk of bias assessed by the Joanna Briggs Institute Critical Appraisal Tools for use in JBI Critical Appraisal Checklist for Analytical Cross-Sectional Studies

Authors	Q1	Q2	Q3	Q4	Q5	Q6	Q7	Q8	% Yes	Risk
Bates and Brown^18^	U	√	√	√	NA	NA	√	√	83.3	Low
McCosker et al.^19^	U	√	√	√	NA	NA	√	√	83.3	Low
Gadomski et al.^20^	--	√	√	√	NA	NA	√	√	83.3	Low
Bender^21^	U	√	√	√	NA	NA	√	√	83.3	Low
Rous and Kurth^22^	√	√	√	√	NA	NA	√	√	100	Low
Durham-Pressley et al.^23^	U	√	√	√	NA	NA	√	√	83.3	Low

Q1 = Were the criteria for inclusion in the sample clearly defined?; Q2 = Were the study subjects and the setting described in detail?; Q3 = Was the exposure measured in a valid and reliable way?; Q4 = Were objective, standard criteria used for measurement of the condition?; Q5 = Were confounding factors identified?; Q6 = Were strategies to deal with confounding factors stated?; Q7 = Were the outcomes measured in a valid and reliable way?; Q8 = Was appropriate statistical analysis used?; √ = Yes; -- = No; NA = Not Applicable; U = Unclear.

### Results of individual studies

Four studies^18–20,23^ provided the percentage of professionals who knew how to identify signs of domestic violence. Five studies^18,20–23^ investigated whether rural healthcare providers had any educational background on violence during their academic careers. Four studies^18,20,22,23^ asked whether professionals screened their patients for signs of violence in clinical practice (**Table 4**).

**Table 4.** Summary of the main results of eligible studies

Authors	Question	Knowledge of reporting requirements (%)	Screen for injuries (%)	Perception of physical indicators (%)	Educational background (%)
Bates and Brown^18^	Health professionals received some training in domestic violence	--	--	--	16
	Under real conditions, health professionals expected that they would be able to recognize victims	--	--	38	--
	Aware of some services to which women could be referred (police or women’s refuge)	12	--	--	--
	Examine alone only when suspecting that the cause of injury was different from what the patient said	--	50	--	--
McCosker et al.^19^	Correctly know the definition of violence against women	--	--	--	25
Gadomski et al.^20^	Had received some past training relative to domestic violence	--	--	--	38
	Had identified a victim in the preceding year	--	39	--	--
Bender^21^	Ask all new patients or all patients periodically about the possibility of abuse and domestic violence	--	49	--	--
	Knowledge of community resources for occasional screening	--	--	--	36
Roush and Kurth^22^	Can recognize victims of intimate partner violence by the way they behave	--	97	--	--
	Know how to ask about the possibility of intimate partner violence and what to do	--	--	--	98
Durham-Pressley et al.^23^	Have sufficient knowledge about familiar violence	--	--	--	38
	Know how to refer patients positive for family violence	64	--	--	--

Bates and Brown^18^ performed a cross-sectional study on physicians and nurses. When asked what kind of injury would raise suspicion of violence, they answered contusion (82%), fractures (58%), and abrasion (38%) and pointed out specific regions of the body, such as injuries to the face (77%). Although only 16% had an educational background on the topic, 38% answered that they would be able to identify signs of domestic violence. Most professionals (90%) agreed that dedicated training would benefit their performance. McCosker et al.^19^ applied a questionnaire before and after a training course on domestic violence and observed a significant change in the knowledge of healthcare providers. A similar strategy focused on training was used by Gadomski et al.^20^ in their eligible study. The authors assessed the knowledge, behavior, and attitudes of professionals and observed improvements in their knowledge of their role as agents to identify violence. The authors also observed that after the training course, healthcare providers were more aware of the importance of referring patients to specialized institutions. When Bender^21^ asked participants about their attitude toward suspicious cases of domestic violence, 16% answered that they would not take any action. The authors observed that the number of hours dedicated to training would increase the likelihood of screening patients for intimate partner violence. Roush and Kurth^22^ observed that most participants had good knowledge and judicious attitudes regarding the identification and management of domestic violence against women. Finally, Durham-Pressley et al.^23^ observed that most professionals (60.9%) had not identified a single case of violence in the last year. Their reported attitude, however, was predominantly correct (63.9%) (**Table 5**).

**Table 5.** Summary of the main results related to attitudes of health professionals of eligible studies

Authors	Referral the victims to specialized agencies (%)	Patient counseling about options (%)	Encourage the victims to leave the violent situation (%)	Confront the victim when she does not admit violence (%)	No action, even identifying cases of violence (%)
Bates and Brown^18^	98	--	67	79	--
McCosker et al.^19^	93	--	--	--	--
Gadomski et al.^20^	46	39	--	--	--
Bender^21^	--	39	--	--	16
Roush and Kurth^22^	48	--	57	--	--
Durham-Pressley et al.^23^	64	--	--	--	--

## DISCUSSION

Violence against women in the rural environment is a multifactorial problem.^2^ Socioeconomic status seems to have an important part in this equation.^24^ Authors have shown subcategories of women who are even more vulnerable to violence in the rural environment, such as the elderly and the unemployed.^25^ More specifically, these women present a major risk of poverty, and their lack of financial independence makes them susceptible to recurrent intimate partner violence.^25^ This is a sole example of the vast casuistics often overlooked about women who live in rural areas. This study contributes evidence-based findings to the scarce scientific literature on this topic.

Healthcare providers normally conduct physical examinations of their patients; thus, it is possible to detect signs of violence through visual inspection. Early studies in the field noticed that contusions, fractures, and abrasions appeared as the most expected signs of physical violence against women when they asked the rural healthcare providers.^18^ Interestingly, most professionals would expect these signs more commonly on the faces of their female patients.^18^ The perception of rural healthcare providers, in this case, was correct and later confirmed by Brink.^26^ These findings raise particular insights, especially regarding the access of healthcare providers to specific anatomic regions of the body. For instance, faces are examined routinely by dentists, speech therapists, otolaryngologists, and ophthalmologists. However, most professionals were not specifically trained to detect violence against women. In a previous systematic review, oral healthcare providers showed an evident lack of educational background on the topic.^11^ It could be speculated, for example, that healthcare providers would receive specialized training in postgraduate studies. It must be noted, however, that the professionals who work in rural areas are not always specialized and have possibly trained for general practice and primary healthcare exclusively.

This systematic review shows that most rural healthcare providers have expressed their interest in specialized training to properly identify and manage cases of violence against women since 1998.^18^ Recent studies in developed countries, such as Australia, have shown that training on the topic of intimate partner violence remains poorly embedded in paramedical undergraduate programs.^27^ When it comes to the specific field of nursery, other authors showed that most training courses are part of an existing program and are not provided as a sole course.^28^ These studies point out a call for a change in the way that training is planned and provided. The positive effects of training were subsequently confirmed by the eligible studies in this systematic review.^19,20^ Most healthcare providers sampled in previous studies were general practitioners;^11^ thus, the strategies developed to implement training must be compatible with their routines, especially in rural areas. During distance training sessions, itinerary training courses conducted throughout the countryside could reach these professionals more easily and be beneficial in transforming their practices. Among the benefits of training sessions is the increased knowledge of how to refer patients with confirmed exposure to violence.^20^ Notably, specific countries impose reports of patients experiencing violence. In Brazil, the Codes of Medical and Dental Ethics, for example, enable the breach of secrecy if justified by the Law. Federal Law n. 10.778/2003 establishes the mandatory report of female patients who are victims of violence and treated in any public or private healthcare institution in the country—including the rural area. In addition to the Brazilian legislation, healthcare providers must expect a transitional scenario of violence against women created by immigrants, especially from neighbors countries in South America. Some immigrants settle in less-expensive cities, such as those in rural areas. Authors have demonstrated that this special group of victims is often marginalized and under-researched;^29^ hence, violence could be even more underreported. They are in the Brazilian territory; thus, reporting suspected cases of violence against women remains mandatory and could shed light on this vulnerable population.

However, reporting remains a persistent issue for healthcare providers. This systematic review shows that the available data are contradictory. On the one hand, recent studies show that most of the professionals (nearly 60%) would undertake the correct attitude and refer the patients to specialized institutions that shelter victims of domestic violence.^22,23^ On the other hand, a considerable amount (16%) of rural healthcare providers would remain silent.^21^ The word “Most,” in these studies, must be carefully interpreted. Despite the majority of correct attitudes among rural healthcare providers in some of the eligible studies, a significant percentage (40%) of professionals still lack knowledge about how to protect female victims of violence. Again, this seems to be a matter of continuing education and preparing for the future. An additional contribution to this scenario would be strategies to increase the victims’ awareness as well as provide them with solutions to self-report domestic violence in a safe environment. The State of São Paulo, in Brazil, for example, had strategies that directly bridged victims and police. In specific, the Police Department developed a “help button” in a smartphone freeware app. Women are invited to register their personal data and activate the button with a single click to provide the police with a GPS signal that reports not only their location but also the situation of imminent violence. Of course, this solution may not uniformly reach rural women. Hence, a call for tailor-made solutions for these women is necessary, and this systematic review is a compilation of evidence to justify strategies with science.

The limitations inherent to this systematic review include the general methodological heterogeneity between eligible articles, which reflects the random approach of authors to design and apply questionnaires. Future studies could focus on developing and validating questionnaires to enable a more standardized research practice and eventually the application of meta-analyses. Additionally, all the eligible studies were only observational and reduced the level of evidence of this systematic review compared to, for instance, reviews of experimental randomized control trials. Overcoming this limitation, however, might be challenging as observational studies might be the most common approach to studying violence against women, while experimental models are not suitable.

## CONCLUSIONS

The screened methodological designs differed considerably among the articles, but, in general, a low risk of bias was detected. Health professionals attending to patients in the rural environment showed restrictions in their knowledge of violence against women, possibly because of a lack of training in the field. Educational training strategies are required for identifying and reporting violence against women in this particular area.
